# Hypericin-Mediated Photodynamic Inactivation Against the Plant Pathogen *Clavibacter michiganensis*: Preventative Seed Decontamination Enhanced by Potassium Iodide

**DOI:** 10.3390/microorganisms13102360

**Published:** 2025-10-14

**Authors:** Linda Jernej, Sonja Gschwendtner, Andreas Kubin, Lionel Wightman, Kristjan Plaetzer

**Affiliations:** 1 Laboratory of Photodynamic Inactivation of Microorganisms, Department of Biosciences and Medical Biology, University of Salzburg, 5020 Salzburg, Austria; linda.jernej@plus.ac.at (L.J.);; 2 HYPERICUM LifeScience GmbH, 1230 Vienna, Austria; a.kubin@hypericum-ls.com (A.K.); l.wightman@hypericum-ls.com (L.W.)

**Keywords:** hypericin, polyvinylpyrrolidone, potassium iodide, agricultural pathogens, photodynamic disinfection, crop protection, white light

## Abstract

*Clavibacter michiganensis* causes significant crop losses in tomatoes, and the disease may be transferred by plant seeds. This study evaluates the efficacy of Photodynamic Inactivation (PDI) with a water-soluble hypericin derivative, developed as a complex with polyvinylpyrrolidone (high hypericin-loaded PVP, HHL-PVP), as a decontamination strategy for tomato seeds. HHL-PVP was chosen for its overall stability, as the complex remains stable in solution for over 950 days, maintains its absorption capacity after illumination with 200 J·cm^−2^, and produces reactive oxygen species (ROS) even at concentrations as low as 1 µM. PDI against *C. michiganensis* with 5 μM HHL-PVP, 10 min drug to light interval (DLI), and illumination with red light (600–700 nm, 100 J·cm^−2^) exceeded the antimicrobial effect of a 99.9% reduction in liquid culture. Increasing the DLI to 24 h did not alter the photokilling effect. A 14 h light/10 h dark cycle in white light (118 J·cm^−2^) with 0.3 µM HHL-PVP inhibited the growth of *C. michiganensis* by more than 6 log_10_ steps, indicating that HHL-PVP has a stable and long-lasting photokilling effect. The combination of HHL-PVP with potassium iodide (KI, 100 mM) completely eradicated *C. michiganensis* in liquid culture with red and white light, indicating KI’s role in enhancing phototoxicity. Tomato seed photodynamic decontamination using 1.0 µM HHL-PVP activated by 200 J·cm^−2^ white light inactivated >5 log_10_ of *C. michiganensis*, without diminishing sprouting. An addition of 100 mM KI increased the percentage of sprouted seedlings and inactivated 100% of bacteria. These results demonstrate that HHL-PVP-mediated PDI combined with KI could be highly effective as a preventative strategy in tomato protection against *C. michiganensis*.

## 1. Introduction

The world’s population is expected to expand to 9.7 billion people by 2050, and with that rises the demand for nutritious food [1,2]. Yet, climate change threatens agriculture through extreme weather events and the inflated abundance of phytopathogens [3]. Due to the lack of sustainable methods, phytopathogens have been reined in by pesticides, leading to a rise in resistance to common antibiotics and fungicides, and with that, a worldwide increase in crop losses [4,5]. Nonetheless, ensuring eco-friendly farmed food for both humans and animals is a central element of the One Health concept. Sustainably farmed crops maintain a healthy ecosystem and thus, fertile grounds. Furthermore, adequate nutrition is essential for proper human and animal growth, immune function, and disease prevention [6].

For many people, tomatoes are a key component to meet a healthy diet. They are rich in essential nutrients such as antioxidants, vitamin C, potassium, lycopene, and folate, and contain crucial fiber [7,8,9]. *Clavibacter michiganensis* subsp. *michiganensis* is a Gram-positive plant pathogen and one of the top 10 bacterial pests, as it causes bacterial wilt in tomatoes [10]. An infected tomato plant will pass on the infection through seeds, on which the bacteria can stick. In addition, adjoining tomato plants can pass biofilm from one vascular system to another via small bruises or hyathodes. The phytopathogen can also survive in cut-down plant material on soil or on close weeds [11,12]. It is estimated that sporadic yield losses of up to USD 300.000 per year for single tomato growers can be expected upon *C. michiganensis* infections [13]. Up to now, no commercially available tomato plant cultivar resistant to *C. michiganensis* infections exists. Outbreaks are, therefore, handled by crop rotation, sanitation measures, or chemical pesticides, which raises concerns about food safety and environmental impact [14].

In recent years, Photodynamic Inactivation (PDI) was considered as an alternative to antimicrobial agents in agriculture and postharvest fruit treatment [15,16,17,18]. PDI uses light-activated photosensitizers (PSs) in the presence of oxygen to generate reactive oxygen species (ROS), such as singlet oxygen or radicals, which cause irreparable oxidative damage to several microbial cell components and thereby can inactivate microorganisms. The risk of resistance development in PDI is minimal, as this process induces destruction on many bacterial sites [19,20]. For agricultural PDI applications, the use of (near-) natural PS, such as chlorophylls, porphyrins, and curcumin or their derivatives, is preferred. These compounds are mostly edible and align with the One Health concept through minimizing chemical residues on crops and in soil. For a successful application of a PS in the field, the substance should be water-soluble, as this ensures simple and even distribution on the plant surface. As most natural PS are inherently hydrophobic, water-soluble analogous, emulsions, or formulations of PS were used previously in agricultural PDI studies. Depending on the application, the PS should be photostable, have a high ROS yield and be easy to handle. Substances with low phytotoxicity are also preferred to maintain food safety [21,22].

Here, we introduce a water-soluble PS-complexation of hypericin as a possible treatment against *C. michiganensis* infections. Hypericin, a natural naphthodianthrone, is mainly found in secondary plant metabolites, especially in *Hypericum perforatum* (St. John’s Wort), and is a common pigment in mushroom species [23,24]. It is a prominent PS in Photodynamic Therapy of cancers and was used as a PS in antimicrobial PDI [25,26]. Hypericin is very photostable but hydrophobic, which means it will aggregate in physiological conditions, resulting in a dramatic reduction in its capability as a PS. To create a water-soluble formulation, hypericin was derivatized as a complex with polyvinylpyrrolidone (PVP, food additive E1201) in order to form hydrophilic vehicles enabling the complete dissolution of the high hypericin-loaded PVP complex (HHL-PVP) in water [27]. The same complexation as used in this study, HHL-PVP, was recently shown to be effective as a PS in Photodynamic Therapy against glioblastoma [28].

This study aims to broaden the application selection of water-soluble HHL-PVP into agriculture as a tomato seed disinfectant. Therefore, we tested the photostability of HHL-PVP under white light and its storage-ability in solution, as well as its ROS production via SOSG and DCFDA assays. Moreover, we determined the photodynamic effect of HHL-PVP in different concentrations against the tomato pathogen *C. michiganensis* in vitro using two different light regimes: (i) illumination with a short period of red light (100 J·cm^−2^, 600–700 nm) or (ii) a long-term illumination in a 24-h cycle (14 h light/10 h dark, 118 J·cm^−2^, white light). Additionally, we spiked HHL-PVP with potassium iodide (KI, 100 mM) in both light regimes. The inorganic salt KI has been shown to act as an enhancer of the photodynamic effect through the reaction of singlet oxygen with water to hydrogen peroxide or iodine radicals, both microbicidal substances [29]. To establish if HHL-PVP with and without KI can be applied as a photodynamic decontamination for tomato seeds, we tested the PS at different concentrations activated by white light (200 J·cm^−2^). The relative inactivation of bacteria on seeds post-PDI and the germination into tomato seedlings were determined.

## 2. Materials and Methods

### 2.1. HHL-PVP Stability Assays and ROS Production

Preparation of the high hypericin-loaded polyvinylpyrrolidone (HHL-PVP) complex was performed as described in European Patent EP3820524B1 [27]. Briefly, hypericin was first dissolved, after which polyvinylpyrrolidone (PVP) was added at a weight ratio of hypericin/PVP = 30:70. The mixture was heated to 210 °C and maintained at this temperature to ensure complete dissolution. After equilibration to room temperature, the resulting amorphous residue was dissolved in water and subsequently freeze-dried.

Photostability experiments were conducted to compare the light-induced degradation of HHL-PVP and protoporphyrin IX (P8293, Sigma-Aldrich Handels GmbH, Vienna, Austria) as porphyrin control; both were dissolved in water at a concentration of 0.25 mg mL^−1^ (equals 148.70 µM of hypericin in solution). For each sample, 20 mL aliquots were placed under a 500 W halogen lamp (DÜWI 93024, düwi GmBH, Breckerfeld, Germany), showing a similar emission spectrum to daylight) at a distance of 40 cm, corresponding to an irradiance of 1 J·cm^−2^ per 40 s. Samples were exposed to light, and aliquots were withdrawn every 30 min. The concentration of each PS was determined spectrophotometrically using a HITACHI U-2001 spectrophotometer (Hitachi, Ltd., 1994, Osaka, Japan), measuring absorbance at 600 nm for HHL-PVP and at 560 nm for protoporphyrin IX. To account for evaporation during irradiation, the samples were weighed before each withdrawal, and the lost volume was replenished with water.

The long-term stability of dissolved HHL-PVP was assessed over a period of 961 days. The powder was weighed (0.21 mg mL^−1^, equals 125.00 µM hypericin in solution), dissolved in water, and the resulting solution was stored in airtight containers at 4 °C until analysis. After various time points, aliquots were withdrawn from the same solution for stability assessment. The soluble hypericin content was quantified by high-performance liquid chromatography (HPLC) and expressed as a percentage by weight of the HHL-PVP complex (Analytical Method for Hypericin; Column ACE 5C18 150 × 4.6 mm, Precolumn Nucleosil 100 5C18, MACHEREY-NAGEL, Altmann Analytik GmbH & Co., KG, München, Germany,, Detection: 588 nm, Injection Volume 20 µL, Flow rate 1.0 mL/min, Mobile PhaseMethanol/Ethylacetate/Phosphatebuffer pH 2). Prior to each stability measurement, calibration curves were generated by dissolving plant-derived hypericin in mobile phase solvent and analyzing samples at various concentrations. Due to fluctuations in calibration conditions and the extended intervals between stability measurements, a new calibration curve was established at each sampling point, and calibration parameters were recalculated accordingly.

The singlet oxygen production of 1.0 µM HHL-PVP in ddH_2_O with and without 100 mM KI was evaluated by Singlet Oxygen Sensor Green Assay (SOSG, Invitrogen by Thermo Fisher Scientific Inc., Waltham, MA, USA), following the method of Hamminger et al. [17]. Briefly, 88.20 µL of the sample was mixed with 1.80 µL of 10 µM SOSG in a 96-well plate. In addition, production of other radical ROS was measured by a 2′,7′-Dichlorofluorescin diacetate assay (DCFDA, Sigma-Aldrich Handels GmbH, Vienna, Austria). The DCFDA was, therefore, reacted to DCFH following the protocol [17]. In a 96-well plate, 87.75 µL of the sample was mixed with 2.25 µL of 10 µM DCFH. For both assays, blanks containing only ddH_2_O with or without 100 mM KI, respectively, were also prepared. Subsequently, all the samples were illuminated with white light (SANSI Plant-Growing Lamp, ISC Lightning Direct, SANSI LED Lightning, INC., Union City, CA, USA, at 655 W m^−2^, 1 J/15 s illumination, absorption spectrum in Supplementary Figure S3) between 0 and 20 J·cm^−2^. Fluorescence signals of SOSG and DCFH were evaluated using an Infinite M200 (Tecan Austria GmbH, Grödig, Austria) microplate reader at excitation wavelength 488 nm and emission at 525 nm (gain set to 70). All the samples were measured in triplicate. The blanks were subtracted from each measurement, and the mean and standard deviation of the fluorescence signals were plotted.

### 2.2. Preparation of Stock Solutions for PDI

HHL-PVP (0.17 mg mL^−1^, 30 *w*/*w*%) was dissolved in ddH_2_O to achieve a stock of 100 µM hypericin in solution. Aliquots were stored in the freezer (−20 °C) until use. For PDI treatments on *C. michiganensis,* the HHL-PVP stock was diluted to give 0.1, 0.3, 1.0, and 5.0 µM of hypericin in DPBS (Sigma Life Science, St. Louis, MO, USA).

A 1 M potassium iodide (KI, Carl Roth GmbH + Co., Karlsruhe, Germany) stock solution was prepared in ddH_2_O and stored in the fridge (4 °C) until use. To evaluate the enhancing efficacy of KI in PDI treatments, the stock was diluted to 100 mM.

Absorption spectra of 100 µM HHL-PVP and 100 µM HHL-PVP plus 100 mM KI were measured using a plate reader Infinite M200 (Tecan Austria GmbH) (Supplementary Figure S1).

### 2.3. Cultivation of Clavibacter michiganensis

The strain *Clavibacter michiganensis* subsp. *michiganensis* DSM 46364 was acquired from the DSMZ (Leibnitz Institute, Braunschweig Germany), and was cryopreserved supplemented with 25% glycerol in liquid nitrogen at −196 °C. Bacterial culture was thawed monthly in 20 mL TSYE medium (30 g L^−1^ CASO-Bouillon (Carl Roth GmbH + Co., Karlsruhe, Germany) and 3 g L^−1^ Yeast Extract (Carl Roth GmbH + Co., Karlsruhe, Germany)), and stored in the fridge (4 °C) until use.

For each experiment, an overnight culture was prepared by incubating 100 µL of bacterial culture in 20 mL TSYE and incubating at 26 °C under constant agitation (200 rpm) for 24 h [30].

### 2.4. HHL-PVP-Mediated Photodynamic Inactivation

To evaluate the photokilling effect of HHL-PVP against *C. michiganensis,* planktonic PDI experiments were performed following the method of Glueck et al. [16] with minor modifications. For experiments using high bacterial load and illumination by the red light lamp, 1.8 mL of the overnight culture were centrifuged at 800 rcf for 3 min. The supernatant was aspirated, and the pellet resuspended in 0, 0.1, 0.3, 1.0, or 5.0 µM HHL-PVP diluted in DPBS. To test the phototoxicity enhancing effect of KI (Carl Roth GmbH), further experiments were performed by spiking these samples with 100 mM KI. Triplicates of 500 µL of each sample were transferred into a 24-well plate (Greiner BioOne, Kremsmünster, Austria). After a drug to light interval (DLI) in the dark of either 10 min or 24 h, the samples were illuminated using a Waldmann PDT 1200 lamp (100 J·cm^−2^, 600–700 nm, Herbert Waldmann GmbH & Co., KG, Villingen-Schwenningen, Germany). The illumination set-up was previously published [31]. Before each experiment, the irradiance was determined using a LI-180 Spectrometer (LI-COR Environmental GmbH, Lincoln, NE, USA), and the time of illumination in seconds was calculated by dividing 100 J·cm^−2^ by the measured irradiance as W·cm^−2^. Duration of illumination for each experiment was approximately 60 min. The emission spectrum was measured using the LI-180 Spectrometer and is given in Supplementary Figure S2A. An additional 24-well plate, which was kept from illumination, containing bacterial suspensions with 0, 1.0 and 5.0 µM HHL-PVP, with and without KI, served as a dark control.

Long-term PDI experiments were performed in a growth chamber (BINDER GmbH, Tuttlingen, Germany, Model KBWF 240, 26 °C, 50% humidity) with a 14 h light/10 h dark cycle. For this, 1 µL of a *C. michiganensis* overnight culture was diluted in 96-well plates (Greiner BioOne, Kremsmünster, Austria) in 200 µL of either 0, 0.1, 0.3, or 1.0 µM HHL-PVP with or without 100 mM KI in DPBS. This ratio of volumes is proportional to the creation of a new overnight culture. The samples were then illuminated for 14 h with a LUMILUX T8 L18W/865 lamp from OSRAM (Inventronics GmbH, Garching b. München, Germany) and subsequently kept in the dark for 10 h. The intensity and spectrum of the lamp were measured using the LI-180 Spectrometer (LI-COR Environmental GmbH, Lincoln, NE, USA) and gave a mean intensity of 23 W m^−2^, resulting in an irradiance of 118 J·cm^−2^ for a 14 h illumination (Supplementary Figure S2B). An additional 96-well plate, which was kept from illumination, containing bacterial suspensions with 0 and 1.0 µM HHL-PVP, with and without KI, served as a dark control.

The results were evaluated by serial dilution of each sample in increments of 10 to reach a final dilution of 10^−7^. Five drops à 10 µL of each dilution were plated on TSYE agar plates (TSYE with 15 g L^−1^ Agar-Agar Kobe I (Carl Roth GmbH + Co., Karlsruhe, Germany)). Plates were incubated at 26 °C for 96 h to count colony-forming units (CFUs). The counted CFUs of each dilution were averaged and extrapolated to a volume of 1 mL for all the samples. The PDI efficacy was visualized as relative inactivation by dividing the CFU mL^−1^ of the double negative control (0 µM, dark; i.e., the detection limit of the experiment) by the respective CFU mL^−1^ of each sample for each biological replicate. All the experiments were replicated three times as independent experiments.

### 2.5. HHL-PVP-Mediated Seed Decontamination

To test if HHL-PVP mediated PDI could be a useful tool to decontaminate tomato seeds from *C. michiganensis* contamination, the experimental procedure of Fellner et al. [32] with minor adaptations was followed. Briefly, organic tomato seeds of the variant Moneymaker (see Supplementary Table S2 for detailed information) were pre-disinfected in 15% H_2_O_2_ (Carl Roth GmbH + Co., Karlsruhe, Germany) for 1 min and subsequently washed in sterile ddH_2_O. Six seeds per treatment were then inoculated in 500 µL overnight culture of *C. michiganensis* (see Section 2.3) for 30 min in a 24-well plate. One bunch of seeds was neither treated nor inoculated and served as sterile control. Then, the seeds were dried in the laminar air flow for 10 min and incubated in 500 µL treatment for a DLI of 10 min. Treatments consisted of 10% Acetic Acid (Carl Roth GmbH + Co., Karlsruhe, Germany) and 1.5% Sodium Hypochlorite (Carl Roth GmbH + Co., Karlsruhe, Germany) solutions diluted in sterile ddH_2_O, serving as controls, and 0 µM, 0.1 µM, or 1 µM HHL-PVP diluted in ddH_2_O with or without 100 mM KI. Illumination was performed under constant agitation using the white-light plant-growing lamp (SANSI Plant-Growing Lamp, ISC Lightning Direct, SANSI LED Lightning, Inc., Union City, CA, USA, at 355 W m^−2^, absorption spectrum in Supplementary Figure S3) with either 100 J·cm^−2^ or 200 J·cm^−2^ (illumination time 47 or 94 min, respectively). Post-illumination, the six seeds per treatment were transferred into 2 mL tubes containing 1 mL DPBS and vortexed for 30 s to get all the bacteria into the solution. Then, the results were evaluated by serial dilution of each sample in increments of 10 to reach a final dilution of 10^−7^. One drop à 5 µL of each dilution were plated on square TSYE agar plates (sample results given in Supplementary Figure S4). The plates were incubated at 26 °C for 96 h. The CFU on the plate were counted to determine the reduction in bacteria and extrapolated to give CFU mL^−1^. The PDI efficacy was visualized as relative inactivation by diving the CFU mL^−1^ of the double negative control (0 µM, dark; i.e., the detection limit of the experiment) by the respective CFU mL^−1^ of each sample for each biological replicate (*n* = 3).

To evaluate if HHL-PVP-mediated seed decontamination impairs germination, 24 tomato seeds per treatment were incubated for 10 min in either 1 mL of 10% Acetic Acid or 1.5% Sodium Hypochlorite, serving as controls, or in ddH_2_O, 1 µM HHL-PVP, or 1 µM HHL-PVP plus 100 mM KI diluted in ddH_2_O. Illumination was performed under constant agitation with the same light regimen as the liquid culture experiments (white light, SANSI Plant-Growing Lamp, ISC Lightning Direct, SANSI LED Lightning, Inc., Union City, CA, USA, at 355 W m^−2^, 200 J·cm^−2^). Then, the seeds were placed in wet soil (SPAR Österr. Warenhandels-AG, Salzburg, Austria, peat-free seedling and herb soil) inside a see-through growth cover and put into the growth chamber at 26 °C and 50% humidity. The germination was monitored after 6 days post planting by counting all the seedlings at the two-leaf stage.

## 3. Results

### 3.1. HHL-PVP Stability Assays

Hypericin is inherently not water-soluble, which limits its agricultural utility. To address this, a derivative was developed by complexing hypericin with polyvinylpyrrolidone (PVP). The final HHL-PVP complex obtained was found to be water-soluble at 40 mg mL^−1^ (equals 23.8 mM of hypericin in solution).

**Figure 1:**
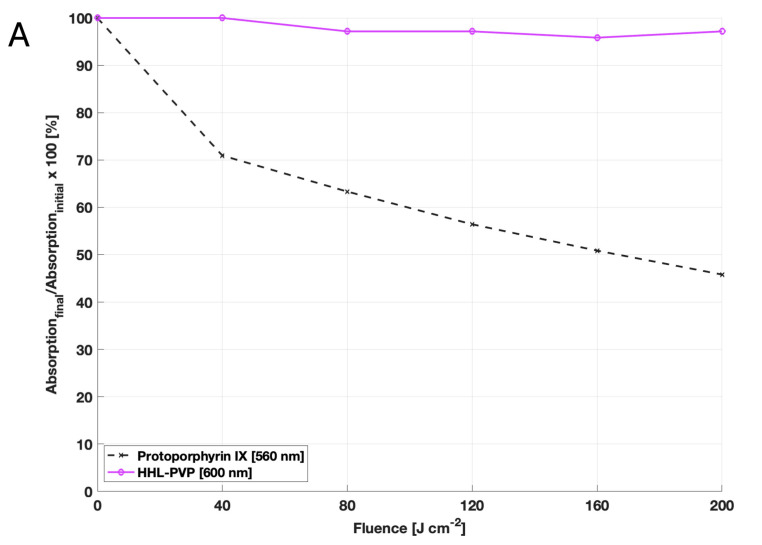

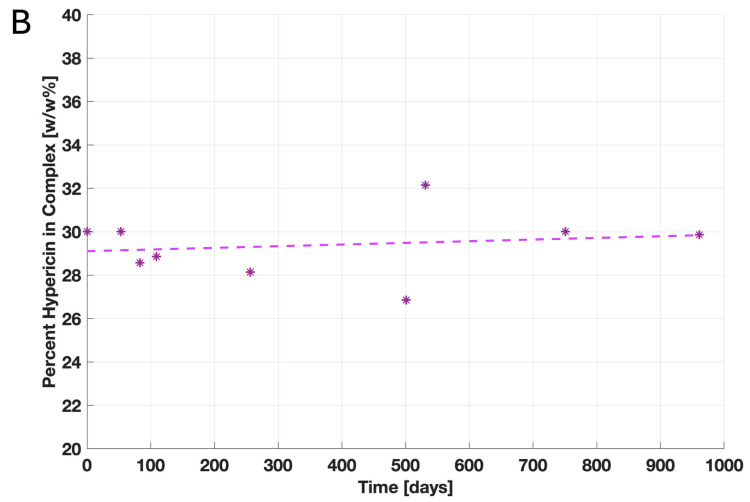
Results of the HHL-PVP stability assays: (**A**) Photostability was evaluated by measuring the decay of the absorption capacity after illumination of protoporphyrin IX at 560 nm and HHL-PVP at 600 nm. Given is the decay of absorption calculated in percentage form, dividing the final absorption capacity by the initial one in the dark. While the absorption of protoporphyrin IX decreases after illumination with 200 J·cm^−2^, HHL-PVP’s absorption barely decays. (**B**) The stability of HHL-PVP as a stock solution stored at 4 °C was measured over 961 days via HPLC. Given is the weight percentage of hypericin in the HHL-PVP complex in weight percentage (*). A trendline (dashed line) between single measurement points indicates that the hypericin percentage is stable at around 30 *w*/*w*%.

For agricultural applications, the storage-ability and stability of a product are as crucial as solubility in water. Over a timeframe of 961 days, the measured content of free hypericin in the HHL-PVP complex did fluctuate negligibly between 26.9 and 32.1 *w*/*w*%. Nonetheless, the stability of the complexation is given when stored cold (Figure 1B). Moreover, photostability was assessed by comparing the bleaching of HHL-PVP to the bleaching of protoporphyrin IX. While the absorption capacity of protoporphyrin IX decreased by more than 50% after illumination, HHL-PVP was determined to be exceptionally photostable (Figure 1A).

**Figure 2:**
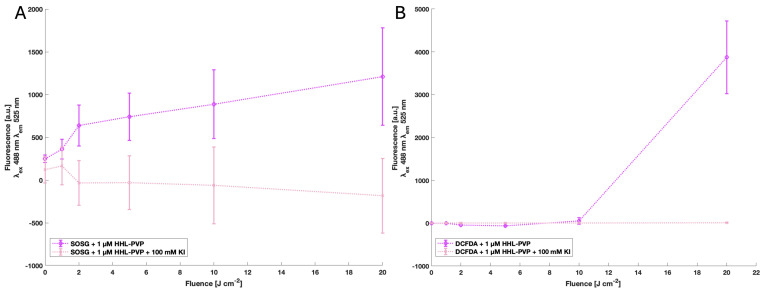
Results of the SOSG assay (**A**) and DCFDA assay (**B**) of 1 µM HHL-PVP and 1 µM HHL-PVP plus 100 mM KI. All the samples were normalized to the blanks ddH_2_O only or ddH_2_O plus 100 mM KI, respectively. Excitation wavelength (λ_ex_) 488 nm; emission wavelength (λ_em_) 525 nm, gain 70. Illuminated up to 20 J·cm^−2^ by a white-light plant-growing lamp. The mean and standard deviation of triplicates are shown. Both ROS types are elevated in the 1 µM HHL-PVP sample, while addition of 100 mM KI quenches the effect of the PS.

The production of both types of ROS, singlet oxygen via the SOSG assay and radicals via DCFDA assay, was identified for 1 µM HHL-PVP, indicating that the substance is a potent photodynamic agent. Addition of the enhancer KI quenched the fluorescence signals of SOSG and DCFDA (Figure 2).

### 3.2. HHL-PVP-Mediated Photodynamic Inactivation

**Figure 3:**
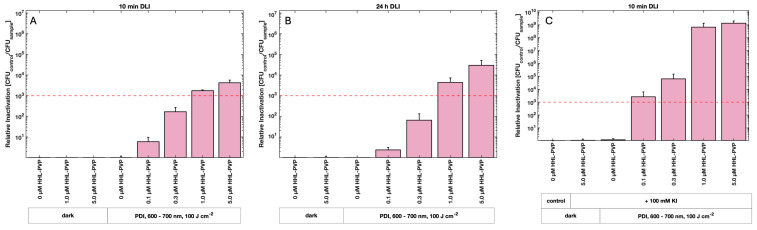
Mean relative inactivation and standard deviation of *Clavibacter michiganensis* after Photodynamic Inactivation using different concentrations of HHL-PVP in the dark or illuminated with 100 J·cm^−2^ of red light (600–700 nm) without (**A**,**B**) or with (**C**) the addition of potassium iodide (KI, 100 mM). Prior to illumination, the samples were kept in the respective treatment solution for either a 10 min drug to light interval (DLI) (**A**,**C**) or a DLI of 24 h (**B**). The red dashed line indicates the antimicrobial effect at a microbial reduction of 3 log10 steps. Biological replicates *n* = 3.

Photodynamic Inactivation using HHL-PVP and a DLI of 10 min against *C. michiganensis* demonstrated a concentration-dependent bactericidal effect (Figure 3A). At 1.0 µM HHL-PVP, a 1.7 × 10^3^-fold inactivation relative to the untreated control (0 µM, dark) was determined. A higher concentration of 5.0 µM HHL-PVP increased the inactivation to 4.2 × 10^3^. Extending the DLI to 24 h of dark incubation before illumination enhanced the reduction only marginally to 4.3 × 10^3^ and 2.9 × 10^4^ for 1.0 and 5.0 µM HHL-PVP, respectively (Figure 3B). *Clavibacter michiganensis* was found to be neither susceptible to red light alone nor to HHL-PVP without illumination. HHL-PVP illuminated under red light (100 J·cm^−2^) is independent of the DLI and exceeds the antimicrobial effect of a 99.9% reduction at a relatively low concentration of 1.0 µM.

To achieve a stronger bactericidal effect, experiments were performed with spiking HHL-PVP with the enhancer potassium iodide (100 mM KI). Treatment of *C. michiganensis* with a DLI of 10 min and a 0.1 µM HHL-PVP in combination with 100 mM KI led to a photoantimicrobial effect, exceeding the threshold of 3-log_10_ steps (Figure 3C). Therefore, only a tenth of the HHL-PVP concentration was found to be effective when spiked with KI compared to HHL-PVP alone. Increasing the concentration to 5.0 µM HHL-PVP spiked with 100 mM KI resulted in a relative inactivation of 1.3 × 10^9^ and therefore reached the detection limit of the assay.

**Figure 4:**
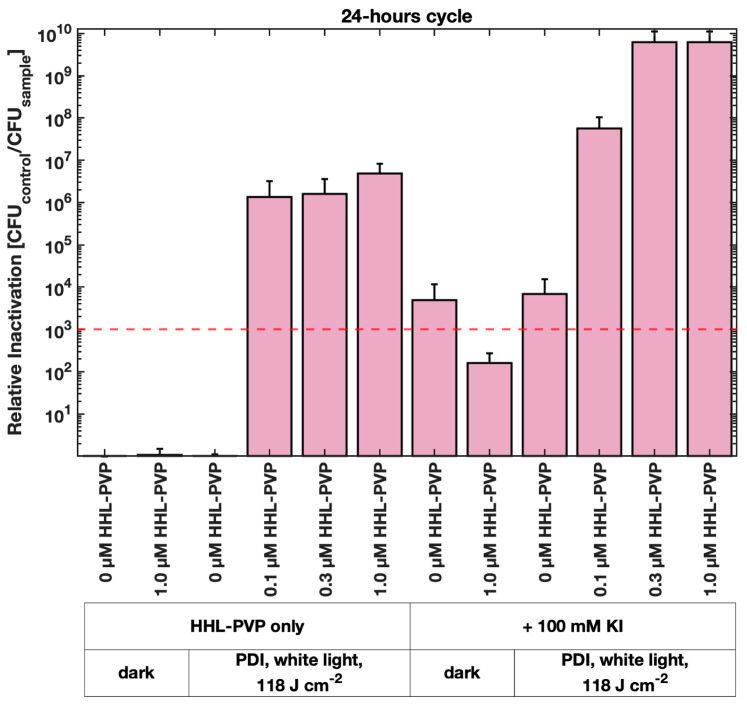
Mean relative inactivation and standard deviation of *Clavibacter michiganensis* after Photodynamic Inactivation using different concentrations of HHL-PVP in the dark or illuminated with 118 J·cm^−2^ of white light in a 14 h light/10 h dark cycle with and without the addition of potassium iodide (KI, 100 mM). The red dashed line indicates the antimicrobial effect at a microbial reduction of 3 log10 steps. Biological replicates *n* = 3.

A long-term PDI experiment was performed to evaluate whether the effect of HHL-PVP on a lower culture load of *C. michiganensis* and under a less powerful white-light regime is similarly effective as red light. For this, 1 µL of bacterial culture was diluted in 200 µL of treatment solution and incubated under white light in a 14 h light/10 h dark cycle, resulting in a total fluence of 118 J·cm^−2^ in 24 h (Figure 4). A relative inactivation of *C. michiganensis* by more than 6 log_10_ steps was induced by 0.1 µM HHL-PVP only. This result surpasses the relative inactivation of 0.1 µM HHL-PVP combined with KI illuminated under red light (Figure 3C). Increasing the concentration further only elevated the relative inactivation slightly. Spiking the HHL-PVP during the 24 h treatment with 100 mM KI inactivated bacteria up to the detection limit at 0.3 and 1.0 µM (eradication of 6.18 × 10^9^ CFU mL^−1^). Nevertheless, 100 mM KI without HHL-PVP already exceeded the antibacterial effect, indicating an inhibitory effect of the salt alone during this long incubation period. Comparable to the experiments under red light illumination, *C. michiganensis* was neither susceptible to the white light alone, nor to HHL-PVP without illumination.

All the results of the HHL-PVP-mediated PDI against *C. michiganensis* are summarized in Supplementary Table S1.

### 3.3. HHL-PVP-Mediated Seed Decontamination

**Figure 5:**
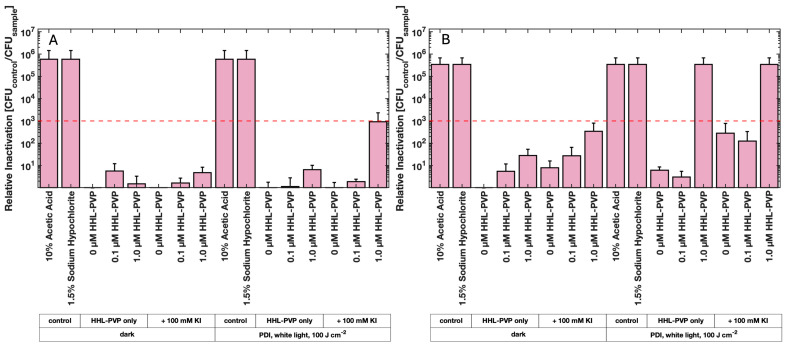
Results of the HHL-PVP-mediated tomato seed photodecontamination. Mean relative inactivation and standard deviation of *Clavibacter michiganensis* after treatment using different concentrations of HHL-PVP with and without the addition of potassium iodide (KI, 100 mM) in the dark or illuminated with white light from a plant-growing lamp at (**A**) 100 J·cm^−2^ (47 min of illumination) or (**B**) 200 J·cm^−2^ (94 min of illumination). 10% Acetic Acid and 1.5% Sodium Hypochlorite served as positive controls. The red dashed line indicates the antimicrobial effect at a microbial reduction of 3 log_10_ steps. Biological replicates *n* = 3.

Tomato seed decontamination experiments were performed to evaluate the in vivo efficiency of the HHL-PVP-mediated PDI (Figure 5). Therefore, tomato seeds were contaminated with *C. michiganensis*, followed by a treatment of either 0.1 µM or 1.0 µM HHL-PVP with and without 100 mM KI and illumination via a plant-growing lamp (white light, 100 or 200 J·cm^−2^, illumination time 47 or 94 min, respectively). Decontamination of the seeds from *C. michiganensis* using the control treatments of 10% Acetic Acid and 1.5% Sodium Hypochlorite resulted in a relative inactivation reaching the detection limit in all the experiments. Activation of 1 µM HHL-PVP by white light with a fluence of 100 J·cm^−2^ could not sufficiently decontaminate seeds. Also, enhancing the effect with 100 mM KI only induced a relative inactivation of 8.99 × 10^2^, merely missing the antibacterial effect (Figure 5A). Doubling the illumination to 94 min (200 J·cm^−2^) increased the decontamination efficiency drastically. HHL-PVP at 1.0 µM inactivated *C. michiganensis* by 3.38 × 10^5^. Combining the PS with 100 mM KI inactivated the pathogens on seeds, reaching the detection limit of 3.41 × 10^5^ (Figure 5B). Nevertheless, similar to the liquid culture experiment with less powerful white light (Figure 4), 100 mM KI has a bactericidal effect close to the antibacterial effect, even without PS.

All the results of the HHL-PVP-mediated seed decontamination are summarized in Supplementary Table S3.

**Figure 6:**
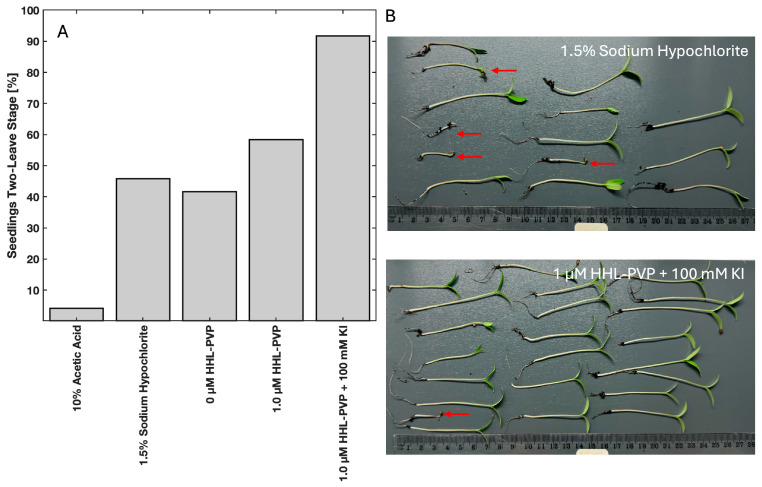
(**A**) Percentage of seedlings that reached the two-leaf stage 6 days after treatment (respective treatment illuminated for 94 min with white light (200 J·cm^−2^)). (**B**) Photographs of the seedlings treated with 1.5% Sodium Hypochlorite or 1 µM HHL-PVP + 100 mM KI. The red arrow indicates seedlings that did not grow up to the two-leaf stage.

The sprouting and seedling development of the HHL-PVP-treated tomato seeds was evaluated by planting the seeds. The tomato seeds were treated with 10% Acetic Acid, 1.5% Sodium Hypochlorite, or ddH_2_O as controls, or with 1 µM HHL-PVP with or without 100 mM KI and illuminated with the plant-growing lamp (white light, 200 J·cm^−2^, 94 min of illumination). After 6 days at 26 °C incubation, 10 of the 24 seeds treated in ddH_2_O grew into seedlings at the two-leaf stage. Eleven of the twenty-four seeds grew after treatment with 1.5% Sodium Hypochlorite. The treatment with 10% Acetic Acid resulted in only 1/24 seedlings, indicating a strong phytotoxicity. While HHL-PVP alone resulted in 14 of 24 seedlings growing to the two-leaf stage, spiking the treatment with 100 mM KI resulted in 22/24 seedlings (Figure 6A). The seedlings treated with 1.5% Sodium Hypochlorite and 1 µM HHL-PVP + 100 mM KI are shown in Figure 6B. The Sodium Hypochlorite treatment resulted in several seeds not reaching the two-leaf stage (red arrows), while only one seedling grew poorly in the HHL-PVP + 100 mM KI-treated group.

A summary of the seed growth experiment and images of all the seedlings are given in Supplementary Table S4 and Supplementary Figure S5.

## 4. Discussion

Although plants make up more than 80% of human nutrition and almost all of livestock feed, plant health is often overseen in the One Health setting [33]. Photodynamic Inactivation is suggested to be one tool that can provide safe and efficient pest management in agriculture, especially when applying natural PS [16,21,22,34]. The PS choice in this study fell on hypericin. Hypericin extracted from St. John’s wort is known for its high singlet oxygen quantum yield in hydrophobic solvents [35]. Yet, for agricultural applications, water solubility is preferred; therefore, a complexation of hypericin to PVP was prepared. Polyvinylpyrrolidone is generally recognized as safe by the U.S. FDA and recognized as a food additive (E1201) in the EU [36,37,38]. There is no evidence of environmental or plant toxicity [39]. Hypericin, on the other hand, is known to have a pharmaceutical impact on humans. It is prescribed as an antidepressant and is a significant choice of PS in Photodynamic Therapy against cancer [40,41]. Photosensitivity after ingestion of high hypericin doses is likely in patients [42]. Yet, there is no data available on phytotoxicity tests using hypericin. For the application as a preventative antibacterial agent on tomato seeds, HHL-PVP was, therefore, tested here.

The long-term illumination (Figure 1A) and storage experiments (Figure 1B) indicate that HHL-PVP ensures consistent efficacy over time, which is crucial for the transport and application of the substance to the field. The ROS production of the HHL-PVP complex was evaluated through the SOSG and DCFDA assays, indicating both a type I and type II photoreaction of the complex (Figure 2). Potent and stable PS-formulations reduce the risk of degradation and have a long-lasting effectiveness. In contrast to curcumin or chlorophyll formulations, both less photostable compounds [43,44], the hypericin derivative might be applied using a different strategy, that is, a bacterial preventative at a very low concentration of HHL-PVP.

Under red light (600–700 nm, 100 J·cm^−2^ applied over approx. 60 min, Figure 3A), 1.0 µM HHL-PVP reaches the antibacterial effect of a 3 log_10_ step reduction against *C. michiganensis* irrespective of the DLI, which points towards a stable and easy-to-use photodynamic system. Under white light with a long exposure (118 J·cm^−2^ applied over 14 h, Figure 4), one tenth of the concentration, 0.1 µM HHL-PVP, inhibits the growth by more than 6 log_10_ steps. We theorize that due to the great photostability of HHL-PVP and the long exposure of bacteria to light and PS, the multiplication of *C. michiganensis* is inhibited. This suggests possible use of long exposure white light, such as daylight or ambient light, and low concentrations of HHL-PVP as a disease prevention strategy.

HHL-PVP enhanced with 100 mM KI inactivated *C. michiganensis* in liquid culture up to the detection limit with red light (600–700 nm, 100 J·cm^−2^) and 5.0 µM HHL-PVP (Figure 3C). With long-term white-light (118 J·cm^−2^) illumination, 0.3 µM HHL-PVP plus 100 mM KI reached the detection limit, yet also a higher toxicity of 100 mM KI without PS was determined (Figure 4). The ROS detection assays showed a quenching of the HHL-PVP photoeffect upon addition of 100 mM KI, indicating that a reaction is occurring (Figure 2). Potassium iodide can react with the produced ROS to peroxyiodide, which further dissociates into free iodine radicals or hydrogen peroxide, both acting bactericidal, explaining the quenching of SOSG and DCFDA signals [43,45,46,47]. The enhancing effect of the inorganic salt in PDI experiments is not new and was shown previously to increase the inactivation of *Escherichia coli*, *Klebsiella pneumoniae*, *Acinetobacter baumanii*, or *Candida albicans* by several log_10_ steps [46,48,49,50,51]. In addition, KI is used as fertilizer in agriculture and was studied as an enrichment in tomato plantations to fight iodine deficiency in the human diet. Yet, KI was shown to be phytotoxic when applied to plants directly [52,53]. As *C. michiganensis* is infiltrating the seeds of infected plants [12], we here tested a PDI protocol for decontaminating the seeds of tomatoes rather than plant material.

**Figure 7:**
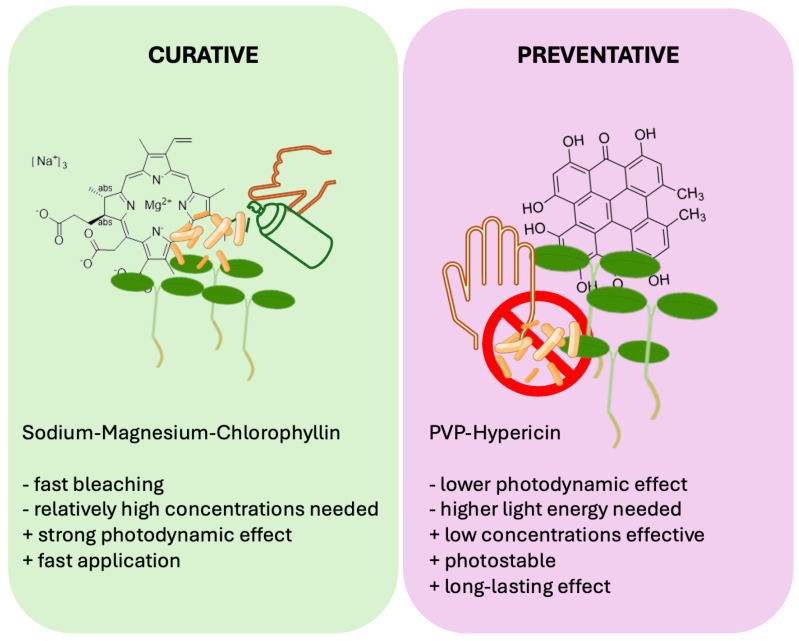
Comparison of two alternative approaches for agricultural Photodynamic Inactivation: curative and preventative treatment. While Sodium–Magnesium–chlorophyllin is a highly effective PS that has a photokilling effect at low light conditions (curative), hypericin derivatives such as HHL-PVP can be applied at very low concentrations and be illuminated for an elongated timespan due to its high photostability (preventative).

Other PDI protocols applying naturally derived PS on seeds or beans revealed that the treatment of spherical objects requires harsher light conditions and higher PS concentrations, compared to liquid cultures. A water-soluble curcumin derivative could inactivate > 3 log_10_ steps of *Escherichia coli* from mung bean surface at 100 µM and 33.8 J·cm^−2^ blue light [54]. Chlorophyllin-based PDI was determined to decontaminate surface-attached microbes by 2.5 log_10_ CFU per gram with 500 µM chlorophyllin and 18 J·cm^−2^ blue light [55]. Chlorophyllin at 100 µM also achieved a relative inactivation of *Listeria innocua* of >3 log_10_ steps on mung beans when activated by blue light. Reduction in bacteria on seeds by PDI could also decrease the bacterial contamination of sprouts [32]. These previous studies used naturally derived PS that are quickly bleached and activated by blue light. We showed that a hundredth of the concentration of the hypericin complex is enough to reach the same effects compared to the previously tested substances. Hypericin-based decontamination has the benefit of activation by red light, the main part of many plant-growing lamps, and the long stability of a low-concentration PS. However, these studies employed light fluences of only a third of the fluences used in this study. Yet, in agricultural applications, when sunlight can be employed, light is not a limiting factor. The liquid culture experiments here indicated that higher fluences of white light over a day/night (24 h) cycle increased the potency of HHL-PVP as a PS without the addition of KI. Therefore, we suggest that instead of a curative approach of using fast-bleaching PS, such as chlorophyllin or curcumin, and low-light, HHL-PVP can be applied as a preventative PS when strong light over a long time is available (Figure 7).

The in vivo application of HHL-PVP-mediated seed decontamination experiments was performed with tomato seeds of the Moneymaker cultivar and 1 µM HHL-PVP. At a protocol with a decreased fluence of 100 J·cm^−2^ (47 min, white-light plant-growing lamp), the enhancer KI is required to achieve a decontamination of 99.9% of *C. michiganensis.* Yet, when doubling the illumination time (94 min, 200 J·cm^−2^), 1 µM HHL-PVP alone inactivated >5 log_10_ steps of bacteria. The addition of 100 mM KI enabled a decontamination up to the detection limit. To test whether the treatment impairs germination, tomato seeds were decontaminated by the same treatment protocol and planted. Acetic Acid (10%) and Sodium Hypochlorite (1.5%) were used as controls, as vinegar and household bleach are commonly used by home gardeners to decontaminate seeds [56]. Both substances impaired the sprouting of tomato seeds into seedlings at the two-leaf stage. Previous studies suggest using lower concentrations, though that reduces the bactericidal effect [56]. HHL-PVP and white light alone did not affect the sprouting. Unexpectedly, the addition of 100 mM KI to the PS even increased the number of seedlings at the two-leaf stage. The potential of KI as a priming agent for tomato seeds might be explained by the compounds produced, such as iodine radicals and hydrogen peroxide. Seed priming is an established method to enhance the seed germination potential and increase stress tolerance of various legumes and fruit plants [57]. Iodine seed priming was proven to have a beneficial effect on tomato and broccoli seedlings by enhancing defense mechanisms and increasing antioxidants [58,59]. Yet, these studies used considerably less concentrated iodine compounds (0.60 mM) than those used in these experiments. The reaction of PS + light + KI induces hydrogen peroxide, as mentioned above. Hydrogen peroxide has the ability to promote germination by interacting with signaling molecules of dormant seeds, yet the exact pathways have not been discovered [60]. Moreover, PDI pre-treatment of seeds introduces a high number of ROS to the dormant system. In Arabidopsis, ROS could trigger sprouting through activation of growth hormone signaling [61]. Hence, formulating a PS with KI for seed decontamination could not only be a suitable method to enhance the phototoxicity towards phytopathogens but might also be a novel technique to increase germination rates. Research should focus on determining growth hormone levels and antioxidant contents of seedlings after KI-enhanced PDI treatment.

HHL-PVP is a robust, ROS-producing, water-soluble, and stable PS with a broad spectrum of possible applications. The natural origin, absorption in visible light, and photodynamic effect at very low concentrations render it a perfect PS for agricultural applications. Low doses of HHL-PVP spiked with 100 mM KI can be applied as a preventative strategy against *C. michiganensis* disease spreading to tomato seeds. A beneficial side-effect of the decontamination with PS plus the enhancer KI could be the fertilizing and seed priming effect of the inorganic salt. While the high KI concentrations showed no phytotoxicity in this study on tomato seeds, further species and application protocols must be tested in order to gain insights into all the possible treatment strategies. Potential feasibility of the treatments under real application conditions must be further studied. Nevertheless, the required light fluences for hypericin activation can economically be reached by using sunlight or readily available plant-growing lamps. Hypericin is still mainly extracted from plant material, but (semi-)synthetic hypericin(-derivatives) were tested, which might decrease the price for the substance [62,63]. Further research should focus on large-scale decontamination experiments on different tomato cultivars and should determine whether phytotoxicity or soil toxicity is possible, as HHL-PVP is highly photostable.

## 5. Conclusions

This study demonstrates that HHL-PVP-mediated Photodynamic Inactivation is an effective strategy for controlling *Clavibacter michiganensis*, particularly when combined with KI as an enhancer. In contrast to fast bleaching photosensitizers, such as chlorophyllin, HHL-PVP might be applied as a preventative disease control due to its properties. The high photostability of HHL-PVP and antibacterial potential under low-energy white light highlight its capability as a preventative bactericide. Further research should focus on optimizing application protocols and elucidating phyto-compatibility. Overall, this work provides a promising foundation for the application of HHL-PVP-mediated PDI in agricultural disease management.

## 6. Patents

Kubin, A., “hypericin-pvp complex with high hypericin content”, European Patent: EP3820524B1.

## Data Availability

The original contributions presented in this study are included in the article/Supplementary Material. Further inquiries can be directed to the corresponding author.

## Supplementary Materials

The following supporting information can be downloaded at https://www.mdpi.com/article/10.3390/microorganisms13102360/s1, Figure S1: Absorption spectra of 100 µM HHL-PVP; Figure S2: Emission spectra of the used light sources; Figure S3: Emission spectra of the used light sources used for HHL-PVP-mediated seed decontamination; Figure S4: Sample results of the HHL-PVP-mediated seed decontamination experiment; Figure S5: All seedlings sprouted after respective treatment; Table S1: Overall average results and standard deviation of the HHL-PVP-mediated PDI experiments; Table S2: Information of used tomato seeds; Table S3: verall average results and standard deviation of the HHL-PVP-mediated decontamination of *C. michiganensis* from tomato seed; Table S4: Results of the sprouting experiment.

## Abbreviations

The following abbreviations are used in this manuscript:

*C. michiganensis*	*Clavibacter michiganenesis*
DCFDA	2′,7′-Dichlorofluorescin Diacetate
ddH_2_O	Double Distilled Water
DLI	Drug to Light Interval
DPBS	Dulbecco’s Phosphate-Buffered Saline
HHL-PVP	High Hypericin-Loaded PVP
HPLC	High-Performance Liquid Chromatography
KI	Potassium Iodide
PDI	Photodynamic Inactivation
PS	Photosensitizer
PVP	Polyvinylpyrrolidone
ROS	Reactive Oxygen Species
SOSG	Singlet Oxygen Sensor Green

## References

[1] United Nations Population Division World Population Prospects 2022: Summary of Results. https://www.un.org/development/desa/pd/content/World-Population-Prospects-2022.

[2] van Dijk M., Morley T., Rau M.L., Saghai Y. (2021). A Meta-Analysis of Projected Global Food Demand and Population at Risk of Hunger for the Period 2010–2050. Nat. Food.

[3] Newbery F., Qi A., Fitt B.D. (2016). Modelling Impacts of Climate Change on Arable Crop Diseases: Progress, Challenges and Applications. Curr. Opin. Plant Biol..

[4] Lucas J.A., Hawkins N.J., Fraaije B.A., Sariaslani S., Gadd G.M. (2015). Chapter Two—The Evolution of Fungicide Resistance. Advances in Applied Microbiology.

[5] Hawkins N.J., Bass C., Dixon A., Neve P. (2019). The Evolutionary Origins of Pesticide Resistance. Biol. Rev..

[6] Yan Z., Xiong C., Liu H., Singh B.K. (2022). Sustainable Agricultural Practices Contribute Significantly to One Health. J. Sustain. Agric. Environ..

[7] Dorais M., Ehret D.L., Papadopoulos A.P. (2008). Tomato (*Solanum lycopersicum*) Health Components: From the Seed to the Consumer. Phytochem. Rev..

[8] Collins E.J., Bowyer C., Tsouza A., Chopra M. (2022). Tomatoes: An Extensive Review of the Associated Health Impacts of Tomatoes and Factors That Can Affect Their Cultivation. Biology.

[9] Li N., Wu X., Zhuang W., Xia L., Chen Y., Wu C., Rao Z., Du L., Zhao R., Yi M. (2021). Tomato and Lycopene and Multiple Health Outcomes: Umbrella Review. Food Chem..

[10] Mansfield J., Genin S., Magori S., Citovsky V., Sriariyanum M., Ronald P., Dow M., Verdier V., Beer S.V., Machado M.A. (2012). Top 10 Plant Pathogenic Bacteria in Molecular Plant Pathology. Mol. Plant Pathol..

[11] Bacterial Canker of Tomato. https://www.ages.at/en/plant/plant-health/pests-from-a-to-z/bacterial-canker-of-tomato.

[12] de León L., Siverio F., López M.M., Rodríguez A. (2011). Clavibacter Michiganesis Subsp. Michiganensis, a Seedborne Tomato Pathogen: Healthy Seeds Are Still the Goal. Plant Dis..

[13] Peritore-Galve F.C., Tancos M.A., Smart C.D. (2021). Bacterial Canker of Tomato: Revisiting a Global and Economically Damaging Seedborne Pathogen. Plant Dis..

[14] Basim H., Basim E., Tombuloglu H., Unver T. (2021). Comparative Transcriptome Analysis of Resistant and Cultivated Tomato Lines in Response to *Clavibacter michiganensis* Subsp. *michiganensis*. Genomics.

[15] do Prado-Silva L., Brancini G.T.P., Braga G.Ú.L., Liao X., Ding T., Sant’Ana A.S. (2022). Antimicrobial Photodynamic Treatment (aPDT) as an Innovative Technology to Control Spoilage and Pathogenic Microorganisms in Agri-Food Products: An Updated Review. Food Control.

[16] Glueck M., Hamminger C., Fefer M., Liu J., Plaetzer K. (2019). Save the Crop: Photodynamic Inactivation of Plant Pathogens I: Bacteria. Photochem. Photobiol. Sci..

[17] Hamminger C., Glueck M., Fefer M., Ckurshumova W., Liu J., Tenhaken R., Plaetzer K. (2022). Photodynamic Inactivation of Plant Pathogens Part II: Fungi. Photochem. Photobiol. Sci..

[18] Jernej L., Liu J., Fefer M., Plaetzer K. (2025). Chlorophyllin and Sunlight against Penicillium Digitatum: Exploring Photodynamic Inactivation as a Green Postharvest Technology in Citriculture. Photochem. Photobiol. Sci..

[19] Natural Photosensitizers in Antimicrobial Photodynamic Therapy. https://www.mdpi.com/2227-9059/9/6/584.

[20] Kashef N., Hamblin M.R. (2017). Can Microbial Cells Develop Resistance to Oxidative Stress in Antimicrobial Photodynamic Inactivation?. Drug Resist. Updates.

[21] Braga G.Ú.L., Silva-Junior G.J., Brancini G.T.P., Hallsworth J.E., Wainwright M. (2022). Photoantimicrobials in Agriculture. J. Photochem. Photobiol. B Biol..

[22] Islam M.T., Ng K., Fefer M., Liu J., Uddin W., Ckurshumova W., Rosa C. (2023). Photosensitizer to the Rescue: In Planta and Field Application of Photodynamic Inactivation Against Plant-Pathogenic Bacteria. Plant Dis..

[23] Kubin A., Wierrani F., Burner U., Alth G., Grunberger W. (2005). Hypericin—The Facts About a Controversial Agent. Curr. Pharm. Des..

[24] Siewert B. (2021). Does the Chemistry of Fungal Pigments Demand the Existence of Photoactivated Defense Strategies in Basidiomycetes?. Photochem. Photobiol. Sci..

[25] Yow C.M.N., Tang H.M., Chu E.S.M., Huang Z. (2012). Hypericin-Mediated Photodynamic Antimicrobial Effect on Clinically Isolated Pathogens. Photochem. Photobiol..

[26] Kiesslich T., Krammer B., Plaetzer K. (2006). Cellular Mechanisms and Prospective Applications of Hypericin in Photodynamic Therapy. Curr. Med. Chem..

[27] Kubin A. (2023). Hypericin-pvp Komplex Mit Hohem Hypericinanteil. European Patent.

[28] Vejzovic D., Kubin A., Fechter K., Karner C., Hartmann J., Ackerbauer T., Radović B., Ritter G., Üçal M., Ropele S. (2025). Glioblastoma Targeting by Water-Soluble Hypericin Derivate HHL-PVP and Photodynamic Tumour Killing. Biomed. Pharmacother..

[29] Vieira C., Gomes A.T.P.C., Mesquita M.Q., Moura N.M.M., Neves M.G.P.M.S., Faustino M.A.F., Almeida A. (2018). An Insight Into the Potentiation Effect of Potassium Iodide on aPDT Efficacy. Front. Microbiol..

[30] Islam M.T., Sain M., Stark C., Fefer M., Liu J., Hoare T., Ckurshumova W., Rosa C. (2023). Overview of Methods and Considerations for the Photodynamic Inactivation of Microorganisms for Agricultural Applications. Photochem. Photobiol. Sci..

[31] Engelhardt V., Krammer B., Plaetzer K. (2010). Antibacterial Photodynamic Therapy Using Water-Soluble Formulations of Hypericin or mTHPC Is Effective in Inactivation of *Staphylococcus aureus*. Photochem. Photobiol. Sci..

[32] Fellner A., Hamminger C., Fefer M., Liu J., Plaetzer K. (2023). Towards Microbial Food Safety of Sprouts: Photodynamic Decontamination of Seeds. Photonics.

[33] Rizzo D.M., Lichtveld M., Mazet J.A.K., Togami E., Miller S.A. (2021). Plant Health and Its Effects on Food Safety and Security in a One Health Framework: Four Case Studies. One Health Outlook.

[34] Liu D., Gu W., Wang L., Sun J. (2023). Photodynamic Inactivation and Its Application in Food Preservation. Crit. Rev. Food Sci. Nutr..

[35] Darmanyan A.P., Burel L., Eloy D., Jardon P. (1994). Singlet Oxygen Production by Hypericin in Various Solvents. J. Chim. Phys..

[36] Nair B. (1998). Final Report On the Safety Assessment of Polyvinylpyrrolidone (PVP). Int. J. Toxicol..

[37] Burnett C.L. (2017). PVP (Polyvinylpyrrolidone). Int. J. Toxicol..

[38] EFSA Panel on Food Additives and Nutrient Sources added to Food (ANS) (2010). Scientific Opinion on the Safety of Polyvinylpyrrolidone-Vinyl Acetate Copolymer for the Proposed Uses as a Food Additive. EFSA J..

[39] Morenko I., Isaeva I., Ostaeva G. (2025). Environmental Aspects of the Use of Water-Soluble Polymers as Stabilizers for Metal Nanoparticles. E3S Web Conf..

[40] Mennini T., Gobbi M. (2004). The Antidepressant Mechanism of *Hypericum perforatum*. Life Sci..

[41] Dong X., Zeng Y., Zhang Z., Fu J., You L., He Y., Hao Y., Gu Z., Yu Z., Qu C. (2021). Hypericin-Mediated Photodynamic Therapy for the Treatment of Cancer: A Review. J. Pharm. Pharmacol..

[42] Couldwell W.T., Surnock A.A., Tobia A.J., Cabana B.E., Stillerman C.B., Forsyth P.A., Appley A.J., Spence A.M., Hinton D.R., Chen T.C. (2011). A Phase 1/2 Study of Orally Administered Synthetic Hypericin for Treatment of Recurrent Malignant Gliomas. Cancer.

[43] Limantara L., Koehler P., Wilhelm B., Porra R.J., Scheer H. (2006). Photostability of Bacteriochlorophyll a and Derivatives: Potential Sensitizers for Photodynamic Tumor Therapy. Photochem. Photobiol..

[44] Chignell C.F., Bilskj P., Reszka K.J., Motten A.G., Sik R.H., Dahl T.A. (1994). Spectral and Photochemical Properties of Curcumin. Photochem. Photobiol..

[45] Mikulich A.V., Plavskii V.Y., Tretyakova A.I., Nahorny R.K., Sobchuk A.N., Dudchik N.V., Emeliyanova O.A., Zhabrouskaya A.I., Plavskaya L.G., Ananich T.S. (2024). Potential of Using Medicinal Plant Extracts as Photosensitizers for Antimicrobial Photodynamic Therapy. Photochem. Photobiol..

[46] Huang L., Szewczyk G., Sarna T., Hamblin M.R. (2017). Potassium Iodide Potentiates Broad-Spectrum Antimicrobial Photodynamic Inactivation Using Photofrin. ACS Infect. Dis..

[47] Li Q., Liu Y., Zheng J., Chen Y., Liu Z., Xie Q., Li D., Xi L., Zheng J., Liu H. (2024). Potassium Iodide Enhances the Killing Effect of Methylene Blue Mediated Photodynamic Therapy against *F. Monophora*. Photodiagnosis Photodyn. Ther..

[48] Reynoso E., Quiroga E.D., Agazzi M.L., Ballatore M.B., Bertolotti S.G., Durantini E.N. (2017). Photodynamic Inactivation of Microorganisms Sensitized by Cationic BODIPY Derivatives Potentiated by Potassium Iodide. Photochem. Photobiol. Sci..

[49] Li Y., Du J., Huang S., Wang S., Wang Y., Lei L., Zhang C., Huang X. (2022). Antimicrobial Photodynamic Effect of Cross-Kingdom Microorganisms with Toluidine Blue O and Potassium Iodide. Int. J. Mol. Sci..

[50] Castro K.A.D.F., Brancini G.T.P., Costa L.D., Biazzotto J.C., Faustino M.A.F., Tomé A.C., Neves M.G.P.M.S., Almeida A., Hamblin M.R., da Silva R.S. (2020). Efficient Photodynamic Inactivation of Candida Albicans by Porphyrin and Potassium Iodide Co-Encapsulation in Micelles. Photochem. Photobiol. Sci..

[51] Wei D., Hamblin M.R., Wang H., Fekrazad R., Wang C., Wen X. (2024). Rose Bengal Diacetate-Mediated Antimicrobial Photodynamic Inactivation: Potentiation by Potassium Iodide and Acceleration of Wound Healing in MRSA-Infected Diabetic Mice. BMC Microbiol..

[52] Ikram N.A., Abdalla M.A., Mühling K.H. (2024). Developing Iron and Iodine Enrichment in Tomato Fruits to Meet Human Nutritional Needs. Plants.

[53] Kiferle C., Gonzali S., Holwerda H.T., Real Ibaceta R., Perata P. (2013). Tomato Fruits: A Good Target for Iodine Biofortification. Front. Plant Sci..

[54] Glueck M., Schamberger B., Eckl P., Plaetzer K. (2017). New Horizons in Microbiological Food Safety: Photodynamic Decontamination Based on a Curcumin Derivative. Photochem. Photobiol. Sci..

[55] Žudyté B., Lukšiené Ž. (2019). Toward Better Microbial Safety of Wheat Sprouts: Chlorophyllin-Based Photosensitization of Seeds. Photochem. Photobiol. Sci..

[56] Gilbert G.S., Diaz A., Bregoff H.A. (2023). Seed Disinfestation Practices to Control Seed-Borne Fungi and Bacteria in Home Production of Sprouts. Foods.

[57] Paparella S., Araújo S.S., Rossi G., Wijayasinghe M., Carbonera D., Balestrazzi A. (2015). Seed Priming: State of the Art and New Perspectives. Plant Cell Rep..

[58] Quispe A.P.V., de Morais E.G., Benevenute P.A.N., Lima J.d.S., dos Santos L.C., Silva M.A., Chalfun-Júnior A., Marchiori P.E.R., Guilherme L.R.G. (2025). Priming Effect with Selenium and Iodine on Broccoli Seedlings: Activation of Biochemical Mechanisms to Mitigate Cold Damages. Plant Physiol. Biochem..

[59] Mejía-Ramírez F., Benavides-Mendoza A., González-Morales S., Juárez-Maldonado A., Lara-Viveros F.M., Morales-Díaz A.B., Morelos-Moreno Á. (2023). Seed Priming Based on Iodine and Selenium Influences the Nutraceutical Compounds in Tomato (*Solanum lycopersicum* L.) Crop. Antioxidants.

[60] Wojtyla Ł., Lechowska K., Kubala S., Garnczarska M. (2016). Different Modes of Hydrogen Peroxide Action During Seed Germination. Front. Plant Sci..

[61] Leymarie J., Vitkauskaité G., Hoang H.H., Gendreau E., Chazoule V., Meimoun P., Corbineau F., El-Maarouf-Bouteau H., Bailly C. (2012). Role of Reactive Oxygen Species in the Regulation of Arabidopsis Seed Dormancy. Plant Cell Physiol..

[62] Huang L.-F., Wang Z.-H., Chen S.-L. (2014). Hypericin: Chemical Synthesis and Biosynthesis. Chin. J. Nat. Med..

[63] Kraus G.A., Pratt D., Tossberg J., Carpenter S. (1990). Antiretroviral Activity of Synthetic Hypericin and Related Analogs. Biochem. Biophys. Res. Commun..

