# Supplementary material for: Hypericin-Mediated Photodynamic Inactivation Against the Plant Pathogen Clavibacter michiganensis: Preventative Seed Decontamination Enhanced by Potassium Iodide
Source: Microorganisms. 2025 Oct 14;13(10):2360. doi: 10.3390/microorganisms13102360 (PMC12565940; doi:10.3390/microorganisms13102360)
Supplement: Supplementary file 1 [file microorganisms-13-02360-s001.zip › microorganisms-3904281-supplementary.pdf]

Supplementary Materials

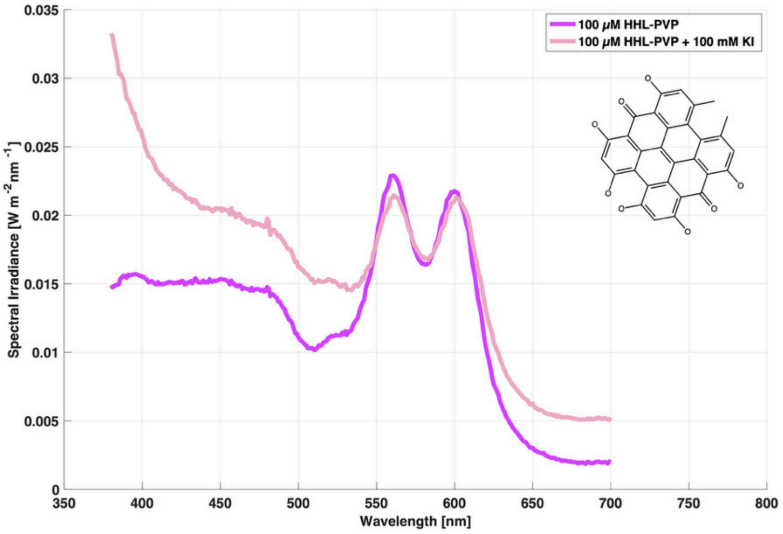

Supplementary Figure S1. Absorption spectra of 100 μM HHL-PVP (PVP-Hyp, dark line) and 100 μM HHL-PVP spiked with 100 mM potassium iodide (KI, light line). The main absorption peaks are only marginally shifted upon addition of KI to HHL-PVP. Given is also the molecular structure of Hypericin.

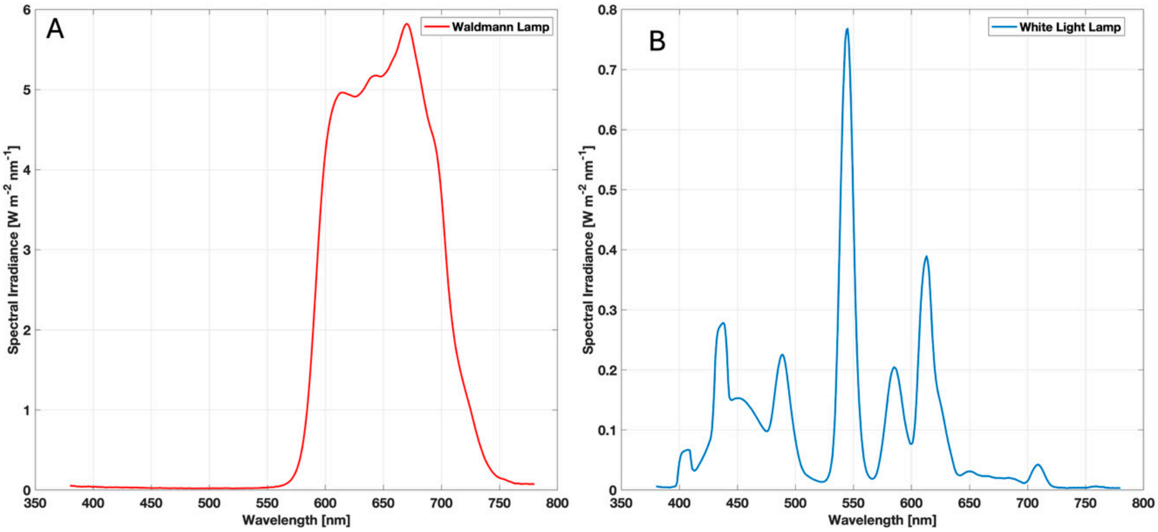

Supplementary Figure S2. Emission spectra of the used light sources in HHL-PVP-mediated PDI for liquid cultures. (A) Spectral irradiance of the Waldmann PDT1200 lamp with main emission at 600 – 700 nm. (B) Spectral irradiance of the white light lamp (LUMILUX T8 L18W/865 lamp from OSRAM (Inventronics GmbH, Germany).

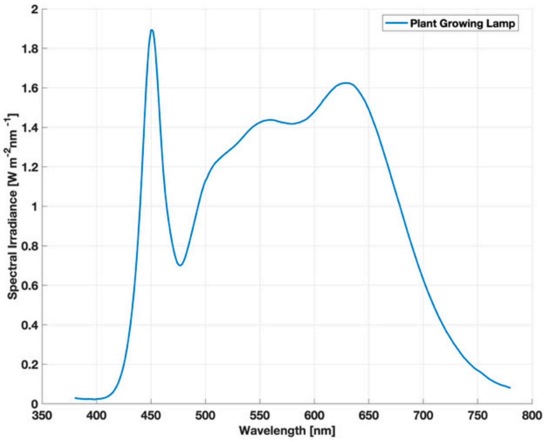

Supplementary Figure S3. Emission spectra of the used light sources used for HHL-PVP-mediated seed decontamination.: white light Plant Growing Lamp (SANSI Plant Growing Lamp, ISC Lightning Direct).

Supplementary Table S1. Overall average results and standard deviation of the HHL-PVP-mediated PDI experiments on *C. michiganensis.* Given are the mean values of CFU mL^{-1} and inactivation values relative to the double negative control (0 µM, dark) for each experiment including respective standard deviations.

	sample	mean CFU mL^{-1}	standard deviation CFU mL^{-1}	mean relative inactivation	standard deviation rel. inactivation
	**10 min DLI**				
**dark**	0 µM	5.83 * 10^08	3.62 * 10^08	1.00 * 10^00	0.00 * 10^00
	1.0 µM	1.79 * 10^09	1.50 * 10^09	4.91 * 10^01	2.58 * 10^01
	5.0 µM	1.63 * 10^09	1.09 * 10^09	3.56 * 10^01	6.90 * 10^02
**PDI, 600-700 nm, 100 J cm^{-2}**	0.1 µM	2.36 * 10^08	2.87 * 10^08	5.82 * 10^00	3.76 * 10^00
	0.3 µM	4.08 * 10^06	2.92 * 10^06	1.65 * 10^02	1.01 * 10^02
	1.0 µM	3.22 * 10^05	1.81 * 10^05	1.74 * 10^03	1.66 * 10^02
	5.0 µM	1.29 * 10^05	4.11 * 10^04	4.18 * 10^03	1.42 * 10^03
	**24 hours DLI**				
**dark**	0 µM	6.80 * 10^08	1.61 * 10^08	1.00 * 10^00	0.00 * 10^00
	5.0 µM	8.03 * 10^08	1.17 * 10^08	8.73 * 10^01	2.37 * 10^01
**PDI, 600-700 nm, 100 J cm^{-2}**	0 µM	8.73 * 10^08	1.76 * 10^08	8.08 * 10^01	1.87 * 10^01
	0.1 µM	3.03 * 10^08	5.04 * 10^07	2.34 * 10^00	7.02 * 10^01
	0.3 µM	2.78 * 10^07	2.03 * 10^07	6.42 * 10^01	6.69 * 10^01
	1.0 µM	9.42 * 10^05	1.46 * 10^06	4.31 * 10^03	2.88 * 10^03
	5.0 µM	2.73 * 10^05	4.48 * 10^05	2.93 * 10^04	2.08 * 10^04
	**10 min DLI**				
**dark**	0 µM	1.33 * 10^09	8.44 * 10^08	1.00 * 10^00	0.00 * 10^00
	5.0 µM + 100 mM KI	1.42 * 10^09	9.96 * 10^08	1.18 * 10^00	2.51 * 10^01

**PDI, 600-700 nm, 100 J cm^{-2}**	0 µM + 100 mM KI	1.27 * 10^09	1.06 * 10^09	1.07 * 10^00	2.53 * 10^01
	0.1 µM + 100 mM KI	1.36 * 10^07	1.40 * 10^07	2.60 * 10^03	3.53 * 10^03
	0.3 µM + 100 mM KI	2.20 * 10^05	2.24 * 10^05	6.40 * 10^04	8.21 * 10^04
	1.0 µM + 100 mM KI	1.37 * 10^02	2.36 * 10^02	6.58 * 10^08	6.76 * 10^08
	5.0 µM + 100 mM KI	0.00 * 10^00	0.00 * 10^00	1.32 * 10^09	6.88 * 10^08
	**24 hour cycle**				
**dark**	0 µM	6.18 * 10^09	5.10 * 10^09	1.00 * 10^00	0.00 * 10^00
	1.0 µM	6.63 * 10^09	5.47 * 10^09	1.08 * 10^00	4.25 * 10^01
**PDI, white light, 118 J cm^{-2}**	0 µM	8.05 * 10^09	3.95 * 10^09	7.62 * 10^01	3.42 * 10^01
	0.1 µM	3.21 * 10^07	4.52 * 10^07	1.35 * 10^06	1.90 * 10^06
	0.3 µM	2.43 * 10^06	3.43 * 10^06	1.58 * 10^06	1.97 * 10^06
	1.0 µM	3.99 * 10^04	5.59 * 10^04	4.92 * 10^06	3.43 * 10^06
**dark**	0 µM + 100 mM KI	1.60 * 10^09	1.13 * 10^09	4.88 * 10^03	6.90 * 10^03
	1.0 µM + 100 mM KI	4.97 * 10^08	6.60 * 10^08	1.60 * 10^02	1.15 * 10^02
**PDI, white light 118 J cm^{-2}**	0 µM + 100 mM KI	2.51 * 10^08	3.53 * 10^08	6.77 * 10^03	8.81 * 10^03
	0.1 µM + 100 mM KI	2.03 * 10^02	1.55 * 10^02	5.67 * 10^07	4.72 * 10^07
	0.3 µM + 100 mM KI	0.00 * 10^00	0.00 * 10^00	6.18 * 10^09	5.10 * 10^09
	1.0 µM + 100 mM KI	0.00 * 10^00	0.00 * 10^00	6.18 * 10^09	5.10 * 10^09

Supplementary Table S2. Information of used tomato seeds in the HHL-PVP-mediated seed decontamination experiments.

**Species**	*Solanum lycopersicum L.*
**Variant**	Moneymaker EC8021
**Country of Origin**	China
**Quality**	Free from GVO
**Vendor**	Germisem Sementes Lda., Portugal (PT 2-3724)
**Batch Number**	3970
**Best Before Date**	01/2028

Supplementary Table S3. Overall average results and standard deviation of the HHL-PVP-mediated decontamination of *C. michiganensis* from tomato seed experiments. Given are the mean values of CFU mL^{-1} and inactivation values relative to the double negative control (0 µM, dark) for each experiment including respective standard deviations.

	**sample**	**mean CFU mL^{-1}**	**standard deviation** CFU mL^{-1}	**mean relative inactivation**	**standard deviation** rel. inactivation
	**47 minutes illuminaton**				
**dark**	10% Acetic Acid	0.00 * 10^00	0.00 * 10^00	5.87 * 10^05	8.57 * 10^05
	1.5% Sodium Hypochlorite	0.00 * 10^00	0.00 * 10^00	5.87 * 10^05	8.57 * 10^05
	0 µM	5.87 * 10^05	6.99 * 10^05	1.00 * 10^00	0.00 * 10^00
	0.1 µM	1.63 * 10^05	1.39 * 10^05	5.65 * 10^00	6.29 * 10^00
	1.0 µM	4.17 * 10^05	2.66 * 10^05	1.49 * 10^00	1.77 * 10^00
	0 µM + 100 mM KI	1.74 * 10^06	1.20 * 10^06	4.23 * 10^-01	3.57 * 10^-01

	0.1 µM + 100 mM KI	3.98 * 10^05	4.62 * 10^05	1.63 * 10^00	1.09 * 10^00
	1.0 µM + 100 mM KI	9.83 * 10^04	7.47 * 10^04	4.75 * 10^00	3.59 * 10^00
**PDI, Plant Growing Lamp, 100 J cm^{-2}**	10% Acetic Acid	0.00 * 10^00	0.00 * 10^00	5.87 * 10^05	8.57 * 10^05
	1.5% Sodium Hypochlorite	0.00 * 10^00	0.00 * 10^00	5.87 * 10^05	8.57 * 10^05
	0 µM	6.92 * 10^05	5.00 * 10^05	9.88 * 10^-01	8.14 * 10^-01
	0.1 µM	2.44 * 10^06	2.87 * 10^06	1.14 * 10^00	1.62 * 10^00
	1.0 µM	8.75 * 10^04	9.74 * 10^04	6.46 * 10^00	3.83 * 10^00
	0 µM + 100 mM KI	1.30 * 10^06	1.73 * 10^06	9.67 * 10^-01	7.73 * 10^-01
	0.1 µM + 100 mM KI	4.47 * 10^05	5.68 * 10^05	1.86 * 10^00	5.68 * 10^-01
	1.0 µM + 100 mM KI	3.85 * 10^03	4.39 * 10^03	8.99 * 10^02	1.39 * 10^03
	**94 minutes illuminaton**				
**dark**	10% Acetic Acid	0.00 * 10^00	0.00 * 10^00	3.41 * 10^05	3.22 * 10^05
	1.5% Sodium Hypochlorite	0.00 * 10^00	0.00 * 10^00	3.41 * 10^05	3.22 * 10^05
	0 µM	3.41 * 10^05	2.63 * 10^05	1.00 * 10^00	0.00 * 10^00
	0.1 µM	8.50 * 10^04	2.47 * 10^04	5.50 * 10^00	6.29 * 10^00
	1.0 µM	4.45 * 10^04	4.64 * 10^04	2.83 * 10^01	2.48 * 10^01
	0 µM + 100 mM KI	3.39 * 10^04	1.82 * 10^04	7.99 * 10^00	7.80 * 10^0
	0.1 µM + 100 mM KI	2.22 * 10^04	1.15 * 10^04	2.68 * 10^01	3.79 * 10^01
	1.0 µM + 100 mM KI	3.21 * 10^03	2.56 * 10^03	3.38 * 10^02	4.42 * 10^02
**PDI, Plant Growing Lamp, 200 J cm^{-2}**	10% Acetic Acid	0.00 * 10^00	0.00 * 10^00	3.41 * 10^05	3.22 * 10^05
	1.5% Sodium Hypochlorite	0.00 * 10^00	0.00 * 10^00	3.41 * 10^05	3.22 * 10^05
	0 µM	7.70 * 10^04	7.85 * 10^04	6.21 * 10^00	2.40 * 10^00
	0.1 µM	8.42 * 10^04	5.25 * 10^04	3.05 * 10^00	2.42 * 10^00
	1.0 µM	3.40 * 10^01	4.67 * 10^01	3.38 * 10^05	3.26 * 10^05
	0 µM + 100 mM KI	3.53 * 10^04	2.93 * 10^04	2.83 * 10^02	4.81 * 10^02
	0.1 µM + 100 mM KI	3.31 * 10^04	2.48 * 10^04	1.22 * 10^02	2.07 * 10^02
	1.0 µM + 100 mM KI	0.00 * 10^00	0.00 * 10^00	3.41 * 10^05	3.22 * 10^05

Supplementary Table S4. Results of the sprouting experiment after treatment with respective substance and illumination at 200 J cm^{-2} with the white light plant growing lamp (94 minutes). The seedlings that reached the two-leaves stage after 6 days of growth at 26 °C were counted. 24 seeds per treatment were planted.

	10% Acetic Acid	1.5% Sodium Hypochlorite	ddH2O only	1 µM HHL-PVP	1 µM HHL-PVP + 100 mM KI
**Seedlings at two-leaves stage**	1	11	10	14	22
**Percentage of seedlings at two-leaves stage**	4.17	45.83	41.67	58.33	91.67

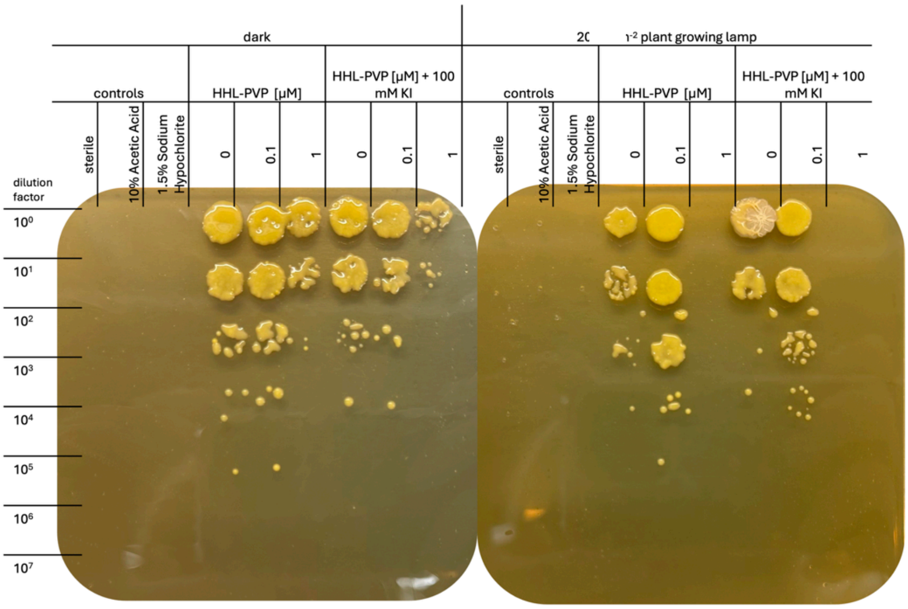

Supplementary Figure S4. Sample results of the HHL-PVP-mediated seed decontamination experiment. Shown are two agar plates with serial dilution of *C. michiganensis* detached from tomato seeds after treatment (dark or illuminated with plant growing lamp at 200 J cm^{-2}).

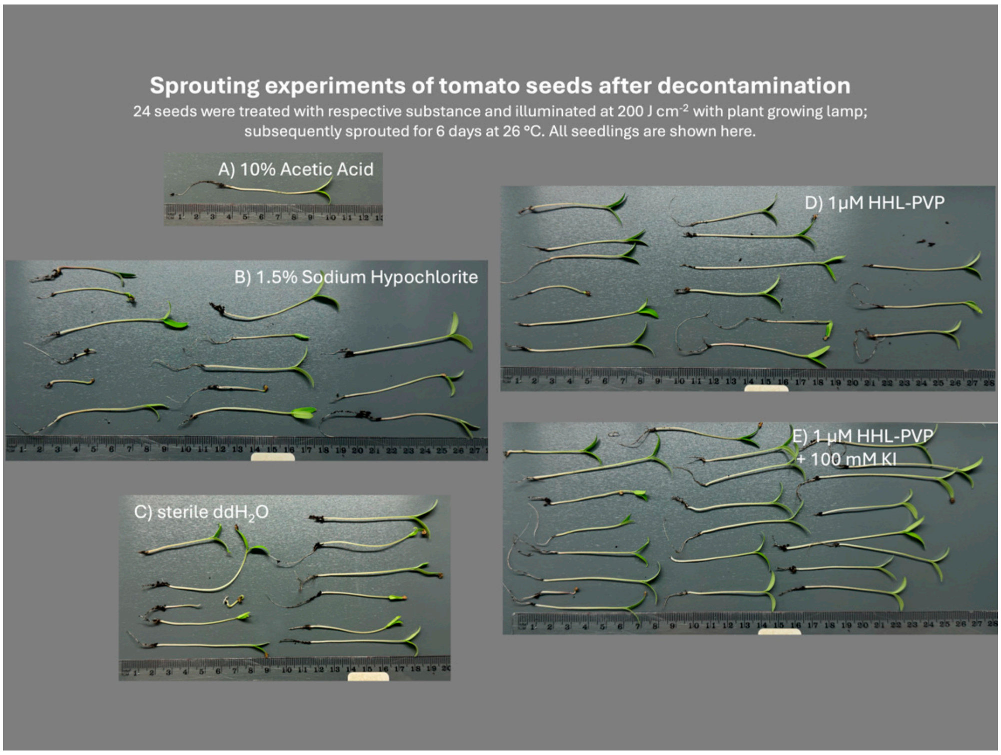

Supplementary Figure S5. All seedlings sprouted after respective treatment.
